# Response of a Wild Edible Plant to Human Disturbance: Harvesting Can Enhance the Subsequent Yield of Bamboo Shoots

**DOI:** 10.1371/journal.pone.0146228

**Published:** 2015-12-31

**Authors:** Noboru Katayama, Osamu Kishida, Rei Sakai, Shintaro Hayakashi, Chikako Miyoshi, Kinya Ito, Aiko Naniwa, Aya Yamaguchi, Katsunori Wada, Shiro Kowata, Yoshinobu Koike, Katsuhiro Tsubakimoto, Kenichi Ohiwa, Hirokazu Sato, Toru Miyazaki, Shinichi Oiwa, Tsubasa Oka, Shinya Kikuchi, Chikako Igarashi, Shiho Chiba, Yoko Akiyama, Hiroyuki Takahashi, Kentaro Takagi

**Affiliations:** 1 Teshio Experimental Forest, Field Science Center for Northern Biosphere, Hokkaido University, Toikanbetsu, Horonobe, Hokkaido, 098–2943, Japan; 2 Tomakomai Experimental Forest, Field Science Center for Northern Biosphere, Hokkaido University, Takaoka, Tomakomai, Hokkaido, 053–0035, Japan; 3 Uryu Experimental Forest, Field Science Center for Northern Biosphere, Hokkaido University, Moshiri, Horokanai, Hokkaido, 074–0741, Japan; 4 Faculty for Educational Research, Nayoro, Field Science Center for Northern Biosphere, Hokkaido University, Tokuda 250, Noyoro, Hokkaido, 096–0071, Japan; Lakehead University, CANADA

## Abstract

Wild edible plants, ecological foodstuffs obtained from forest ecosystems, grow in natural fields, and their productivity depends on their response to harvesting by humans. Addressing exactly how wild edible plants respond to harvesting is critical because this knowledge will provide insights into how to obtain effective and sustainable ecosystem services from these plants. We focused on bamboo shoots of *Sasa kurilensis*, a popular wild edible plant in Japan. We examined the effects of harvesting on bamboo shoot productivity by conducting an experimental manipulation of bamboo shoot harvesting. Twenty experimental plots were prepared in the Teshio Experimental Forest of Hokkaido University and were assigned into two groups: a harvest treatment, in which newly emerged edible bamboo shoots were harvested (n = 10); and a control treatment, in which bamboo shoots were maintained without harvesting (n = 10). In the first year of harvesting (2013), bamboo shoot productivities were examined twice; i.e., the productivity one day after harvesting and the subsequent post-harvest productivity (2–46 days after harvesting), and we observed no difference in productivity between treatments. This means that there was no difference in original bamboo shoot productivity between treatments, and that harvesting did not influence productivity in the initial year. In contrast, in the following year (2014), the number of bamboo shoots in the harvested plots was 2.4-fold greater than in the control plots. These results indicate that over-compensatory growth occurred in the harvested plots in the year following harvesting. Whereas previous research has emphasized the negative impact of harvesting, this study provides the first experimental evidence that harvesting can enhance the productivity of a wild edible plant. This suggests that exploiting compensatory growth, which really amounts to less of a decline in productivity, may be s a key for the effective use of wild edible plants.

## Introduction

An ecological system provides a variety of valuable services for human wellbeing, and these benefits have been called “ecosystem services.” Ecosystem services are sustainable for as long as the ecosystem providing them functions stably. However, disturbances, especially anthropogenic disturbances, can alter the state of the ecosystems [1], sometimes with intense impacts result in a dramatic loss of ecosystem services via catastrophic regime shifts [2,3]. Although it is commonly recognized that disturbances lead to the degradation of ecosystem services, it has also been suggested that some disturbances could improve ecosystem services [4,5]. In order to receive benefits effectively as well as sustainably from ecosystems, we need to address our lack of a comprehensive knowledge about the varying impacts of disturbances on ecosystem services.

Wild edible plants are provisioning ecosystem services obtained from a variety of ecosystems [6]. Throughout human history, humans have identified edible plants from many species of plants in their local environments, and the edible plants have been essential food resources for living, especially in early human history and in times of self-sufficient lifestyles. Even today, many people around the world enjoy gathering and eating wild edible plants. However, the collection of wild edible plants (i.e., harvesting) can be regarded as a human (anthropogenic) disturbance to those plants and their ecosystems. In many cases, harvesters tend to collect plants from the same habitats annually. It is thus important to understand how harvesting influences the subsequent properties (such as demography and succession) of wild edible plants, so that information can be gathered regarding the sustainable use of ecosystem services. Simply stated, we need to know how collecting wild edible plants influences the subsequent plant yield.

To address this issue, it is necessary to study the impacts of harvesting on wild edible plants by using our knowledge of fundamental ecology. Over the past two decades, plant responses to disturbance have been a central focus in plant and community ecology research, and there is a growing body of evidence suggesting the profound impacts of disturbance on the growth and chemical composition of plants [7,8], with consequences for ecosystem functioning [9–11]. Among the various plant responses to disturbance, compensatory plant growth is a widespread phenomenon, in which plants produce new vegetative organs in response to tissue damage [7,12,13].

Plants are likely to undergo compensatory growth if they have at least one of three major characteristics: (1) abundant resource storage [14], (2) rapid growth ability with a high photosynthetic rate [14], and (3) one or more physiological processes that facilitate resource reallocation [15]. New tissues produced by compensatory growth are more flexible and contain a greater amount of nitrogen than normal tissues that promote plant growth. Ecologists have demonstrated that compensatory growth has a role in plant tolerance against negative impacts of herbivory [16,17], and it has also been recognized that compensatory growth provides new resources to herbivorous organisms [10,18]. It is also possible that harvesting by humans can enhance the productivity of wild edible plants by inducing compensatory growth. We tested this hypothesis in the present study by conducting a field experiment mimicking the standard method of harvesting wild edible plants in nature.

The focus of our study was bamboo shoots of *Sasa kurilensis*. Asian peoples customarily eat newly emerged bamboo shoots, and *S*. *kurilensis* is a popular species of edible bamboo. It is a dominant understory species in North Japanese forests, covering over 17% of the forest area [19]. Genetically identical individuals (genets) of *S*. *kurilensis* spread quickly over a wide area due to the plant’s high vegetative growth ability and the extension of numerous shoots (ramets) [20]. These shoots are connected with each other via horizontal underground stems (rhizomes), and they exchange nutrients among themselves [21,22]. We thus hypothesized that the compensatory growth of *S*. *kurilensis* could be induced by harvesting, because it displays the characteristics of plants in which compensatory growth can potentially be induced, i.e., resource storage, rapid growth ability, and capacity for resource re-allocation.

In this study, we assessed bamboo shoot productivity by experimentally manipulating bamboo shoot harvesting in the Teshio Experimental Forest of Hokkaido University, Japan. We enlisted individuals with a great deal of bamboo shoot harvesting experience to collect the bamboo shoots following the criteria by which common harvesters collect bamboo shoots (i.e., the harvesters collect bamboo shoots until they cannot easily find good shoots). In the harvesting year and the following year, we measured the number of bamboo shoots produced at the sites where bamboo shoots had been harvested and at sites where bamboo shoots had been maintained without harvesting, and we examined whether compensatory growth occurred after harvesting. In previous studies examining the effects of harvesting on wild edible plants, researchers consistently showed that harvesting reduced the productivity of the plants by decreasing their abundance [23–26]. The present study is the first to report a scenario in which harvesting was observed to enhance the productivity of wild edible plants.

## Materials and Methods

### Materials

*Sasa kurilensis* is an understory dwarf bamboo species (typical height: 1.5–3 m) that grows in northern mountainous regions in Japan (S1 Appendix). The lifespan of individual shoots is approx. 8 years [27]. After the snowmelt (i.e., early June in northern mountainous regions in Japan), new *S*. *kurilensis* shoots emerge around the matured shoots. These newly emerged shoots are harvested for human consumption within a few days of emergence because they grow and stiffen rapidly, making them inedible. Since the bamboo shoots have a unique taste, they are very popular as a consumable wild vegetable in Japan.

### Study site

This study was conducted in the Teshio Experimental Forest of Hokkaido University (45°03’N, 142°07’E), which is located in the northernmost Japanese forest zone. The mean annual temperature in this forest is 5°C (max. 35°C, min. −35°C), and the annual precipitation is approx. 1000 mm. About one half of the region’s annual precipitation occurs as snowfall between late November and early April. Because forest fires occurred several times during the last 100 years and customary forest practices have resulted in open spaces that promote bamboo invasion, bamboo bushes cover wide areas in the forests.

### Field experiment

We carried out the field experiment to examine the effects of harvesting on bamboo shoot productivity by mimicking the standard method of bamboo shoot collection in the wild. We established 20 experimental plots (10 × 10 m^2^) along a forest road in late May 2013. The plots were >10 m apart from each other. We assigned 10 of the plots to the “harvest treatment” group in which bamboo shoots were collected following the method described below, and the other 10 plots were assigned as the “control treatment” group in which bamboo shoots were not collected. To minimize confounding effects arising from location, we alternatively assigned the harvest and control treatments among the sites (see S1 Appendix). Around the center of each of the plots, we established a 2 × 2-m^2^ quadrat to measure the numbers of bamboo shoots (edible, newly emerged shoots) and bamboo grass (non-edible, matured shoots). We used the densities in the quadrats as representative values of the corresponding plots.

We invited expert harvesters (aged 25 to 64) to collect bamboo shoots in the harvest treatment plots in early June 2013. During the short time that is suitable for harvesting the bamboo shoots, multiple groups of people enter the bamboo bush areas to collect bamboo shoots from several sites. To mimic this situation, we formed two groups consisting of four members, and they collected bamboo shoots from the harvest treatment plots two times, on June 6th and 10th, 2013. Generally, harvesters collect bamboo shoots moderately instead of exhaustively at each site, because they move to other sites as soon as they cannot easily find the edible bamboo shoots. In this study, we asked our harvesters to collect bamboo shoots based on this ordinary harvesting policy in the plots, and we recorded the number of shoots taken from each quadrat. All of the harvesters were aware that their collections were being performed as part of a scientific study.

Because we did not know exactly when the bamboo shoots emerged, we conducted surveys three times. At 1, 18 and 46 days after the second harvesting (i.e., on June 11th and 28th and July 26th, 2013, respectively), we counted the remaining (non-harvested) young bamboo shoots in the plots. Since new bamboo shoots did not emerge after late June (see the Results), we finished our counting by the third survey (on July 26th, 2013). We calculated bamboo shoot productivity as the sum of the number of harvested and leftover bamboo shoots. Note that we included remaining withered shoots because they could be suitable for consumption at the time of their emergence, and because the wasted production (e.g., production of dead organs) is usually included in evaluations of productivity.

To compare bamboo shoot productivity between treatments, we measured the number of bamboo shoots in the control plots on the same days that we counted the remaining bamboo shoots in the harvested plots (on June 11th and 28th and July 26th, 2013). We also counted the number of culms of bamboo grass in each quadrat to evaluate bamboo density, which potentially has effects on the productivity of bamboo shoots, on June 28th 2013.

To evaluate how harvesting influences bamboo shoot productivity and the density of bamboo grass in the year following harvesting, we evaluated bamboo shoot productivity and the density of bamboo grass in 2014, using the same method as in 2013. We harvested bamboo shoots from harvested plots on May 30th and June 4th 2014, and counted the numbers of produced bamboo shoots and bamboo grass in all experimental quadrats on July 7th 2014.

### Statistical analysis

We counted the number of leftover bamboo shoots a total of three times (1, 18 and 46 days after harvesting) in 2013, but for logistical reasons (i.e., very tight schedule), we could not check the number of pre-harvest bamboo shoots. Instead, as an index of pre-harvest productivity, we used the sum of the number of harvested bamboo shoots and the remaining shoots one day after the harvest. We think that this index is reasonable because compensatory growth in bamboo is not likely to occur within such a short time (see Discussion). In addition, we calculated the subsequent productivity of bamboo shoots one day after the first survey (on June 11th, 2013) by subtracting the number of bamboo shoots obtained in the first survey from that in the third survey (on July 26th, 2013). Hereafter, we call the former and latter data the ‘productivity one day after harvesting (from June 11th, 2013)’ and the ‘subsequent post-harvest productivity (from June 12th and July 26th, 2013),’ respectively. Note that the data from the second survey (on June 28th, 2013) were not used in the following analyses, except to check whether shoots continued to be produced.

To examine whether over-compensatory growth occurred in 2013, we separately compared the productivity one day after harvesting and the subsequent post-harvest productivity using *t*-tests. If there was no difference in the productivity one day after harvesting between treatments, and if the subsequent post-harvest productivity in the harvested plots was greater than that in the control plots, we conclude that over-compensatory growth had occurred.

To examine how harvesting influenced the bamboo shoot survival in 2013, we counted the number of surviving bamboo shoots (i.e., the number of leftover shoots excluding the number of withered shoots) until July 26th 2013, and we calculated the survival ratio as the number of surviving shoots divided by the total number of produced shoots (i.e., overall bamboo shoot productivity). The survival ratio was subject to arcsine-square-root transformation. Subsequently, we compared these two values (i.e., the number of surviving shoots and the survival ratio) between treatments using *t*-tests.

To examine whether bamboo grass density is indicative potential bamboo shoot production, we performed a linear regression between the density of bamboo grass and the overall bamboo shoot productivity in 2013, in which the harvesting had less impact on the both bamboo shoot productivity and bamboo grass density. Because the overall bamboo shoot productivity was highly correlated with bamboo grass density (see Results), we compared the densities of bamboo grass between treatments to check whether there was any difference in the potential bamboo shoot production between treatments at the start of our experiment.

Next, we compared the bamboo shoot productivity in 2014 (the year following the harvesting) between treatments by *t*-test to judge whether over-compensatory growth had occurred. Despite the careful setting-up of the experimental plots, the overall bamboo shoot productivity in 2013 (i.e., the original productivity) in the harvest treatment was slightly higher compared to that in the control treatment, although the difference was not significant (see Results). To correct for this difference we conducted two additional analyses. The first analysis is an analysis of covariance (ANCOVA) using ‘bamboo shoot productivity in 2013’ as a covariate in order to account for differences in original productivity. For another correction, we calculated the ‘relative productivity’ by dividing the bamboo shoot productivity in 2014 by that in 2013 in individual plots. In this analysis, we log-transformed the ‘relative productivity’ for standardization (i.e., we calculated the ‘log response ratio’), and we compared it between treatments by *t*-test. To examine how the density of bamboo grass changed after harvesting, we compared the density of bamboo grass in 2014 between treatments, using ‘the density of bamboo grass in 2013’ as the covariate.

## Results

### Harvest and survival ratios

**Fig 1:**
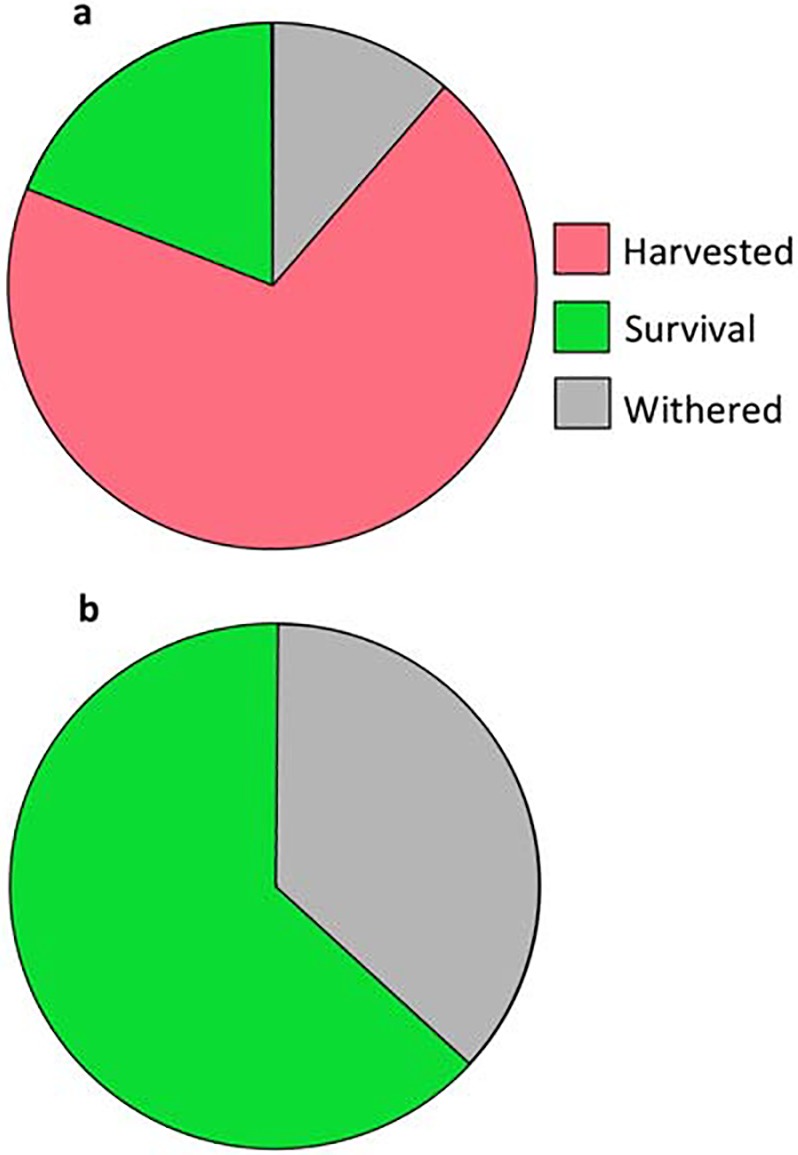
Numerical proportion of harvested (red area), survival (green area) and withered (gray area) bamboo shoots in 2013. (a) Harvest treatment and (b) control treatment.

We harvested 66% of the bamboo shoots that were produced in the harvested plots in 2013 (Fig 1A). Because a fraction of the leftover bamboo shoots withered due to herbivory by insects or rats, 24% and 60% of the remaining shoots survived to July 26th 2013 in the harvest and control groups, respectively (Fig 1). The survival ratio in the harvested plots was significantly lower than that in the control plots (*t* = 2.21, *P* = 0.040), and harvesting decreased the number of surviving bamboo shoots by 42% (the number of surviving shoots [mean ± SE] shoots/m^2^; control treatment: 1.67 ± 0.35, harvest treatment: 0.67 ± 0.34; *t* = 2.03, *P* = 0.058).

### Productivity in the year of harvesting

**Fig 2:**
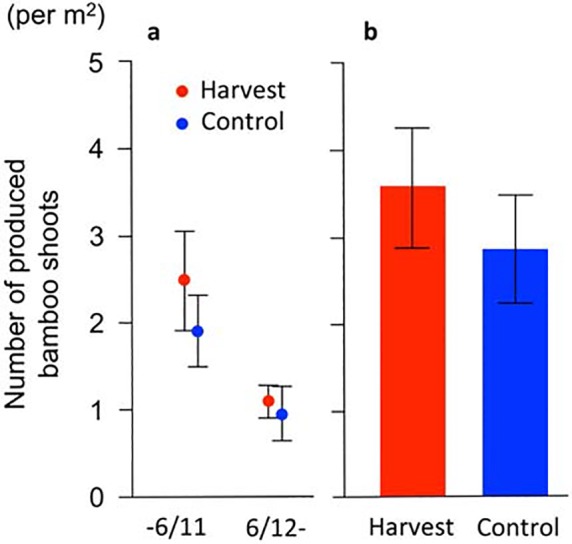
Productivity of bamboo shoots in the year of harvesting. (a) Numbers of produced bamboo shoots one day after harvesting (on June 11th, 2013) and 2–46 days after harvesting (from June 12th to July 26th 2013). Red circles: harvest. Blue circles: control. (b) Total number of bamboo shoots produced in 2013. Bars: SE values.

We observed that a total of 256 bamboo shoots were produced in the 20 quadrats in 2013. The majority of them sprouted up in June, and 68% of the bamboo shoots produced in 2013 had emerged by June 11th; only four bamboo shoots emerged after June 28th. There were no significant differences in the number of bamboo shoots produced by one day after harvesting (on June 11th) and subsequent post-harvest surveys (from June 12th to July 26th) between treatments (one day after harvesting: *t* = −0.80, *P* = 0.43; 2–46 days after harvesting: *t* = −0.34, *P* = 0.74, Fig 2A). Consequently, the total number of bamboo shoots produced in 2013 did not differ between treatments (*t* = 0.74, *P* = 0.47, Fig 2B).

**Fig 3:**
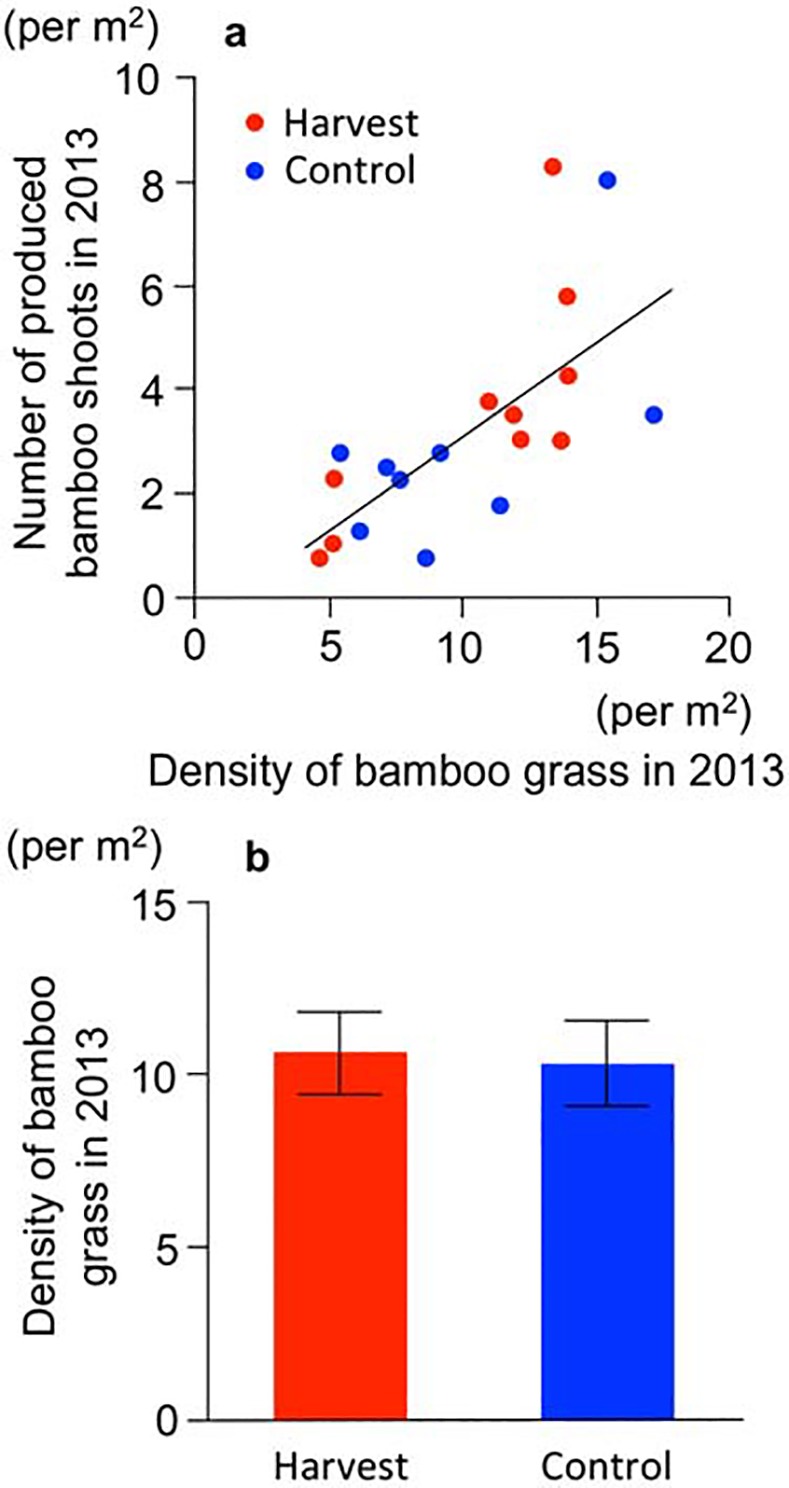
Initial status of bamboo grass in the year the experiment was started (2013). (a) Relationship between the density of the matured bamboo grass and the bamboo shoot productivity. Red circles: harvest. Blue circles: control. Solid line indicates the linear regression between the density of matured bamboo grass and bamboo shoots. (b) Density of matured bamboo grass. Bars: SE values.

A positive relationship existed between bamboo shoot productivity and the density of bamboo grass in 2013 (*R* ^2^ = 0.46, *P* < 0.001, Fig 3A). This means that the higher density of bamboo grass produced more local bamboo shoots. In addition, the density of the bamboo grass did not differ between treatments (*t* = −0.17, *P* = 0.87, Fig 3B), suggesting that the potential productivity of the bamboo shoots did not differ between treatments at the start of this experiment.

### Productivity following harvesting

**Fig 4:**
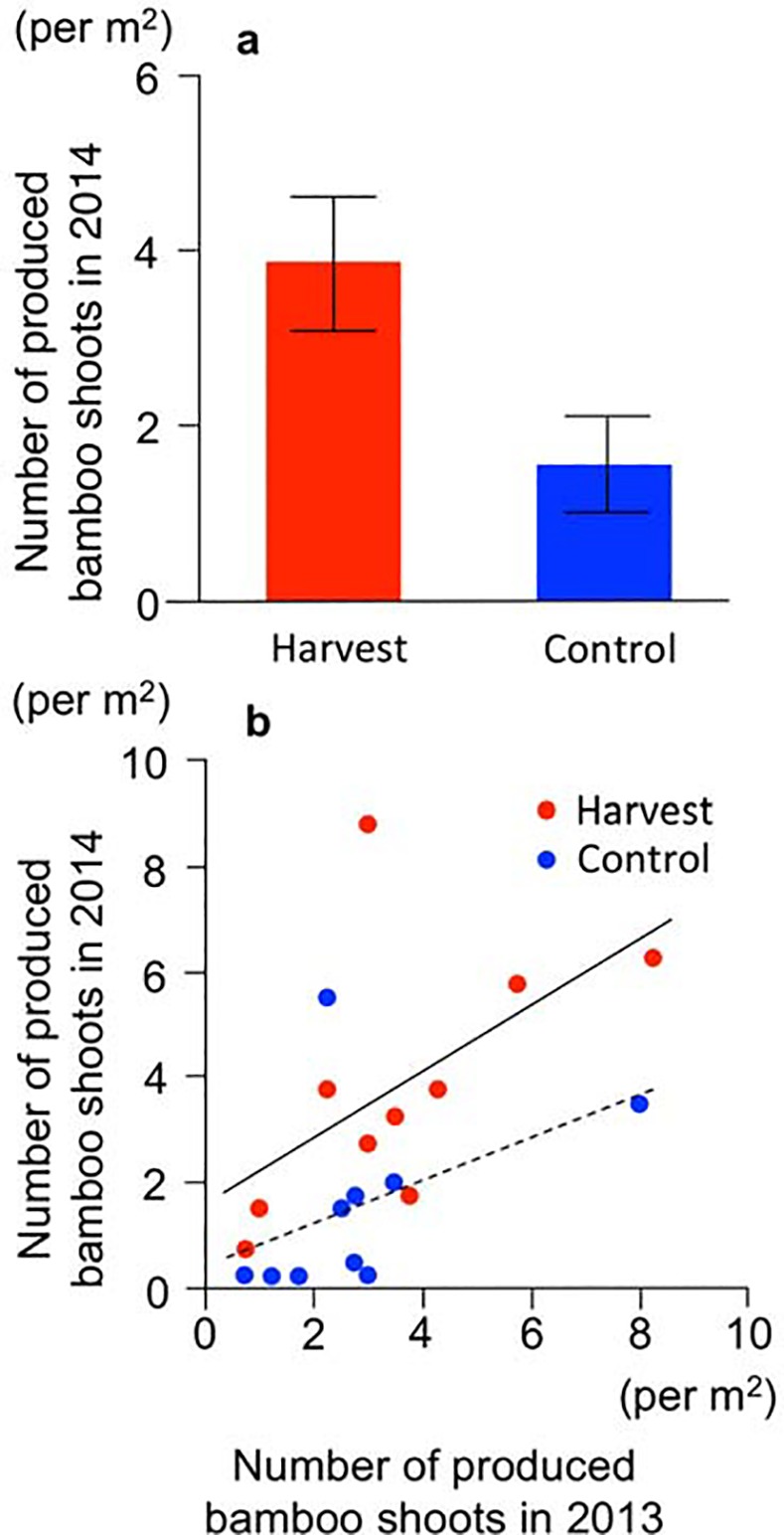
Productivity of bamboo shoots in the year following harvesting (2014). (a) Numbers of bamboo shoots produced. Bars: SE values. (b) Relationship of produced bamboo shoots between the year of harvesting (2013) and the next year (2014). Red circles: harvest. Blue circles: control. Solid and dashed lines indicate the linear regression of the harvest and control treatment, respectively.

In 2014, a total of 216 bamboo shoots were observed in the 20 quadrats. The number of bamboo shoots produced in 2014 differed significantly between treatments (*t* = −2.36, *P* = 0.030), and the harvesting in 2013 enhanced the bamboo shoot productivity in 2014 by 2.4-fold (Fig 4A).

The ANCOVA incorporating ‘bamboo shoot productivity in 2013’ as a covariate revealed a positive correlation between the numbers of bamboo shoots in 2013 and 2014 (*F*_*1*,*1*_ = 5.61, *P* = 0.031, Fig 4B) and a significant effect of treatment on bamboo shoot productivity in 2014 (*F*_*1*,*1*_ = 4.70, *P* = 0.046, Fig 4B), but no interactive effect between the number of bamboo shoots in 2013 and the treatments (*F*_*1*,*1*_ = 0.29, *P* = 0.60).

Although bamboo shoot productivity in 2014 was lower than that in 2013 throughout the sites, the trend of bamboo shoot production differed between treatments. In the control treatment group, the number of bamboo shoots produced in 2014 was 55% lower than that in 2013, whereas bamboo shoot productivity slightly increased in the harvested group (corresponding to 108% of the 2013 productivity). Consequently, the relative bamboo shoot productivity in the harvest treatment was significantly greater than that in the control treatment (*t* = −3.20, *P* = 0.005, S2 Appendix).

### Bamboo grass density

**Fig 5:**
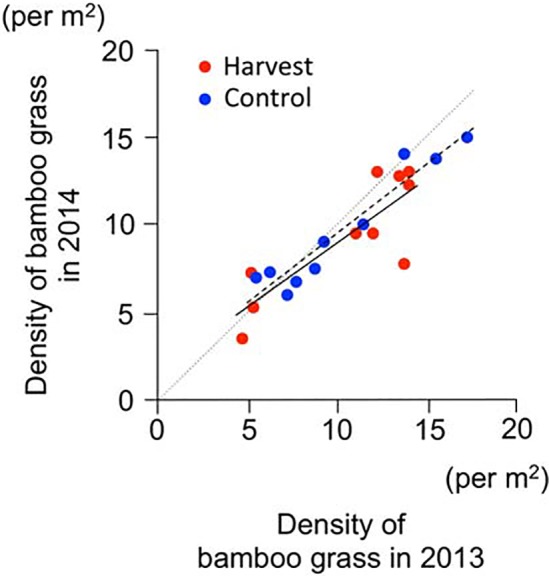
Change bamboo grass density after harvesting. Red circles: harvest treatment. Blue circles: control treatment. Solid and dashed lines indicate the linear regression of the harvest and control treatment, respectively. Dotted line denotes no change in bamboo grass density between 2013 and 2014.

The density of bamboo grass in 2014 was significantly higher in the plots in which the density in 2013 was higher (*F*_*1*,*1*_ = 69.02, *P* < 0.001, Fig 5). However, we did not observe a significant effect of harvesting in 2013 on the density of the bamboo grass in 2014 (*F*_*1*,*1*_ = 0.47, *P* = 0.50) or on the change in density of bamboo grass (this is shown as the ‘interaction term’) (*F*_*1*,*1*_ = 0.16, *P* = 0.69).

## Discussion

In this field experiment, we observed no difference in the bamboo shoot productivity between the harvest and control treatments in 2013, the initial year of harvesting (Fig 2). Moreover, the number of bamboo shoots that emerged after harvesting in that year (i.e., the bamboo shoots that emerged from 12th June to 26th July 2013) did not differ significantly between the treatments (Fig 2A), and thus compensatory growth did not occur in the initial year of this study. However, in the following spring (in 2014), the number of bamboo shoots in the harvested plots was 2.4-fold greater than that in the control plots (Fig 4A).

To conservatively evaluate the effects of harvesting, we performed an ANCOVA to correct for the difference in original productivity between treatment plots. In this model, harvesting also had a positive effect on bamboo shoot productivity (Fig 4B). Similarly, we found a positive effect of harvesting on relative bamboo shoot productivity (S2 Appendix). We therefore conclude that over-compensatory growth occurred in harvested plots in the year following harvesting. Our findings thus provide the first experimental evidence that harvesting (a human disturbance) can enhance the productivity of wild edible plants.

Bamboos have high photosynthetic rates [28], and they can also repair tissue loss by transporting resources from other ramets via underground stems [22]. These capabilities may promote compensatory growth. In the present study, bamboo productivity was enhanced in the year following harvesting, but not in the year of harvesting. This might be due to the phenology of bamboo shoot production. Compensatory plant growth depends on aspects of phenology such as the timing of sprouting, branching, and tillering [13,29]. *Sasa kurilensis* organizes overwintering sprouts from June to September, some of which grow into new shoots the next year [30]. Therefore, it takes about one year to produce new bamboo shoots, and consequently the compensatory growth may not occur in the initial year of harvesting. In our experiment, we harvested bamboo shoots in early June when the overwintering sprouts started to be formed, and thus it is plausible that the compensatory growth was induced in the bamboo in response to the stimulus during the sprout-organization period.

Plants exhibit a variety of responses other than compensatory growth that are induced by disturbances. For example, plants accumulate secondary metabolic substances or stiffen their tissues in response to insect or mammalian herbivory [7,10], responses that help prevent subsequent herbivory by reducing performance and/or the preference of herbivores [31,32]. Bamboo shoots contain silica [33] and secondary metabolic substances such as oxalic acids [34] and homogentisic acids [35], and these substances determine food texture and bitter and astringent tastes. In this study, we investigated neither the chemical compounds nor the taste of bamboo shoots, and thus further studies are necessary to evaluate the effects of harvesting on the qualities of bamboo shoots as food.

### How does long-term harvesting influence bamboo shoot productivity?

Although we observed that short-term harvesting promoted bamboo shoot productivity in the year following harvesting, there is a further question to be addressed: will the enhancement of bamboo shoot productivity be maintained even after long-term harvesting (i.e., harvesting for two or more years in a row)? The induction of compensatory growth depends on the availability of resources in plants [14,36], and compensatory growth is less likely to occur when the plants contain smaller amounts of resources. The balance between the amount of photosynthetic assimilation and the total loss of catabolism and exploitation determines the amount of plant resource storage. Thus, compensatory growth cannot be maintained in bamboo if the loss of resource due to harvesting is greater than the amount of resource assimilation over a long period of time.

In addition, long-term harvesting may influence bamboo shoot productivity by changing the density of bamboo grass. The productivity of bamboo shoots depends on the density of bamboo grass (Fig 3A) because the bamboo grass has organs to produce overwintering sprouts [20,27]. Although our present data indicated no change in the density of bamboo grass for one year after harvesting (Fig 5), it is possible that long-term harvesting could inhibit the recruitment and density of bamboo grass given that harvesting has a strong effect on bamboo shoot survival (Fig 1).

Based on the two hypothetical processes described above, we might expect long-term harvesting to lower bamboo shoot productivity. However, the expert harvesters in our study area felt that based on their experiences, greater numbers of bamboo shoots emerge at sites where the harvesters have annually collected them compared to sites where they rarely visit. Several characteristics of bamboo, such as its high photosynthetic rate and resource reallocation, may prevent the reduction of resource storage and bamboo density. We need to investigate how long-term harvesting affects bamboo productivity in future research.

### Is it rare that harvesting enhances plant productivity?

Over the past few decades, a dramatic decline has been observed in the abundance and habitats of several wild edible plants, e.g., American ginseng (*Panax quinquefolius L*.), goldenseal (*Hydrastis canadensis* L.), and ramps (*Allium tricoccum* Aiton). Along with the growing concern about the sustainable use and conservation of wild edible plants, several researchers demonstrated that harvesting suppressed the abundance of and genetic variation in wild edible plants [23,24,26]. Rock et al. [24] monitored the population dynamics of *A*. *tricoccum* after harvesting, and they estimated that when 25% of *A*. *tricoccum* shoots are harvested, it would take approximately 22 years for the populations to recover to their initial states. Thus, previous studies have consistently pointed out the negative impacts of harvesting on wild edible plants, and a positive impact has never been reported, to our knowledge.

Compensatory growth may be induced in response to harvesting even among the numerous wild edible plants that are pioneer species exhibiting high disturbance tolerance [6]. This is supported by the observation that wild edible plants produce new tissues after fires or floods [6]. We speculate that the reason why no study has reported a positive impact of harvesting is the method used to collect the plants: for the majority of the wild edible plants that were the focus of the previous studies, the roots or whole plant bodies are used as food, and thus harvesters (and researchers) collected the plants by digging up the roots. It is possible that compensatory growth does not occur after such a destructive collection method.

In addition, there are several requirements for the induction of compensatory growth such as the presence of a great amount of resources [14], high photosynthetic capacity [37], and resource reallocation [15]. Clonal pioneer plants such as bamboo tend to satisfy these conditions, and consequently compensatory growth in response to harvesting is more likely to be induced in these plants compared to other plant groups.

In contrast to vegetables planted in artificial environments, wild edible plants grow in natural fields, and the productivity of wild edible plants depends on their response to harvesting. Although the present study examined whether compensatory growth is one of the key mechanisms to enhance the productivity of wild edible plants by focusing on harvesting as a disturbance, artificial manipulations may also induce compensatory growth. For example, the cutting of matured shoots could be another disturbance that induces compensatory growth in wild edible plants [7]. If this is the case, making forest roads through bushes of wild edible plants that exhibit high disturbance tolerance might be an effective way to induce compensatory growth. This may also be true for searching for and collecting the edible shoots, which could lead to greater compensatory growth. Understanding how human disturbances induce compensatory growth could suggest guidelines for the efficient use of wild edible plants, and our present findings contribute information about how we can sustainably use ecosystem services, i.e., wild edible plants, from forest ecosystems.

## Supporting Information

S1 Appendix Photographs of bamboo and map of study site. (a) Matured bamboo grass (*Sasa kurilensis*) and (b) its young edible shoot. The height of a person in (a) is about 170 cm. (c) Location of study site in the Teshio experimental forest. Solid curve in (c) denotes a forest road. Solid and open squares indicate “harvest” and “control” research plots (10 × 10 m), respectively.
(PDF)

S2 Appendix Relative productivity of bamboo shoots. The relative productivity was the log-transformed proportion of shoot productivity in 2014 divided by that in 2013 for the same plot. Bars: SE values.
(PDF)
