# Supplementary material for: Response of a Wild Edible Plant to Human Disturbance: Harvesting Can Enhance the Subsequent Yield of Bamboo Shoots
Source: PLoS One. 2015 Dec 31;10(12):e0146228. doi: 10.1371/journal.pone.0146228 (PMC4697856; doi:10.1371/journal.pone.0146228)
Supplement: S1 Appendix — (a) Matured bamboo grass (Sasa kurilensis) and (b) its young edible shoot. The height of a person in (a) is about 170 cm. (c) Location of study site in the Teshio experimental forest. Solid curve in (c) denotes a forest road. Solid and open squares indicate “harvest” and “control” research plots (10 × 10 m), respectively. (PDF) [file pone.0146228.s001.pdf]

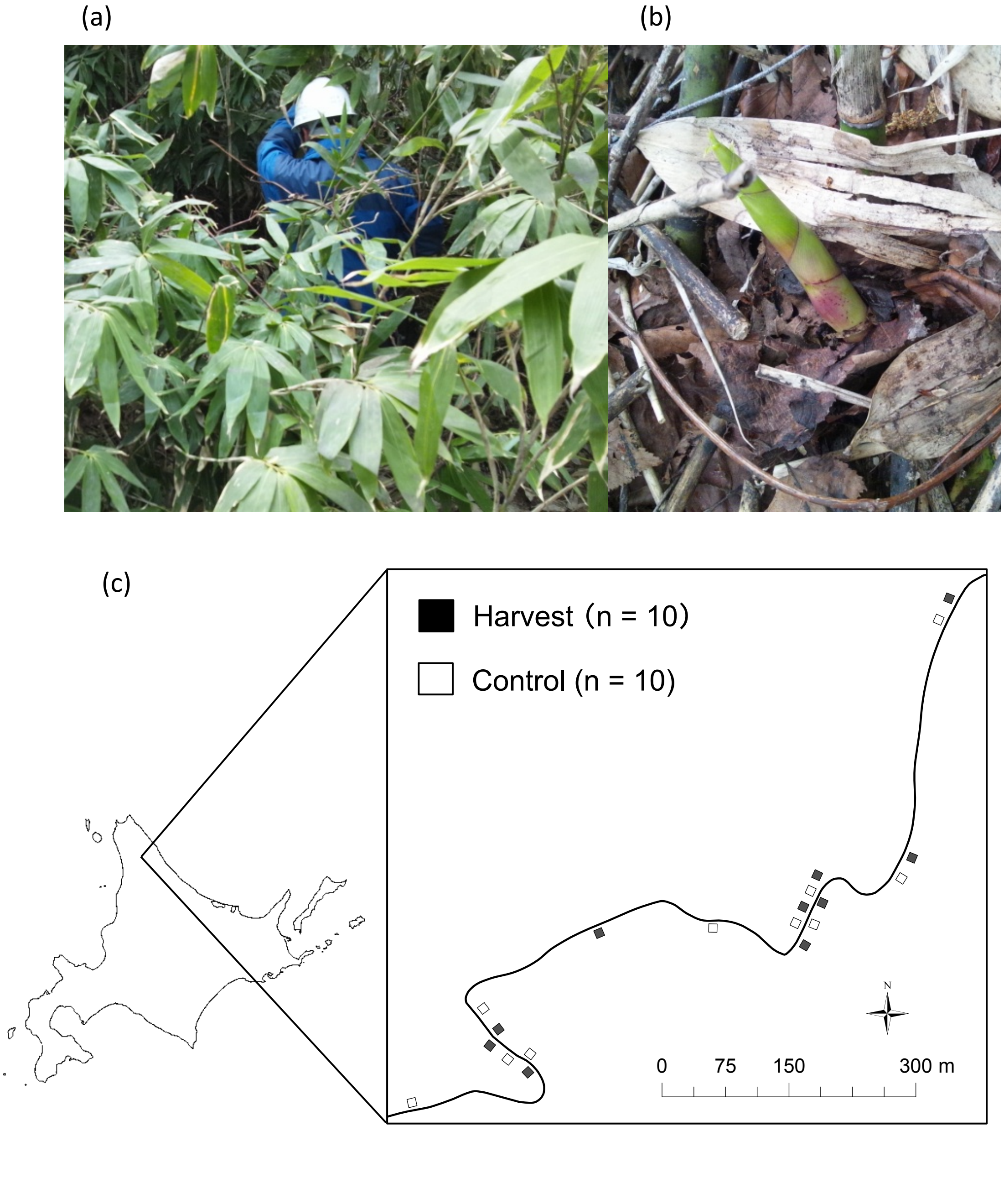

S1 Appendix. Photographs of bamboo and map of study site. (a) Matured bamboo grass (*Sasa kurilensis*) and (b) its young edible shoot. The height of a person in (a) is about 170 cm. (c) Location of study site in the Teshio experimental forest. Solid curve in (c) denotes a forest road. Solid and open squares indicate "harvest" and "control" research plots (10 × 10 m), respectively.
