# Supplementary material for: Response of a Wild Edible Plant to Human Disturbance: Harvesting Can Enhance the Subsequent Yield of Bamboo Shoots
Source: PLoS One. 2015 Dec 31;10(12):e0146228. doi: 10.1371/journal.pone.0146228 (PMC4697856; doi:10.1371/journal.pone.0146228)
Supplement: S2 Appendix — The relative productivity was the log-transformed proportion of shoot productivity in 2014 divided by that in 2013 for the same plot. Bars: SE values. (PDF) [file pone.0146228.s002.pdf]

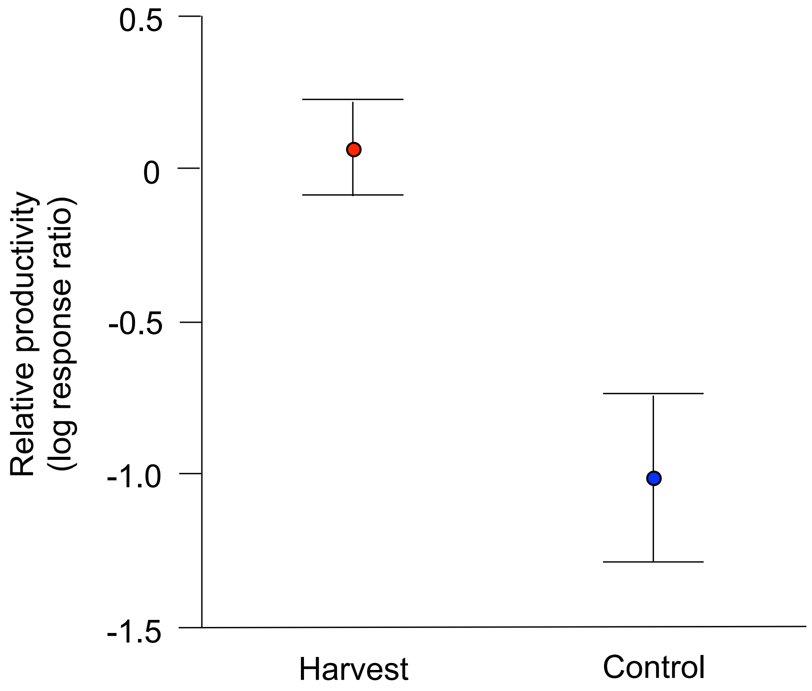

S2 Appendix. Relative productivity of bamboo shoots. The relative productivity was the log-transformed proportion of shoot productivity in 2014 divided by that in 2013 for the same plot. Bars: SE values.
